# Microbial lysates repurposed as liquid egg substitutes

**DOI:** 10.1038/s41538-024-00281-y

**Published:** 2024-06-19

**Authors:** Kyeong Rok Choi, Da-Hee Ahn, Seok Yeong Jung, Yu Hyun Lee, Sang Yup Lee

**Affiliations:** 1 Metabolic and Biomolecular Engineering National Research Laboratory, Systems Metabolic Engineering and Systems Healthcare Cross-Generation Collaborative Laboratory, Department of Chemical and Biomolecular Engineering (BK21 four), Korea Advanced Institute of Science and Technology (KAIST), Daejeon, 34141 Republic of Korea; 2 BioProcess Engineering Research Center, KAIST, Daejeon, 34141 Republic of Korea; 3 BioInformatics Research Center, KAIST Institute for the BioCentury, KAIST Institute for Artificial Intelligence, KAIST, Daejeon, 34141 Republic of Korea; 4 Graduate School of Engineering Biology, KAIST, Daejeon, 34141 Republic of Korea; 5 Present Address: Research and Development Center, GS Caltex Corporation, Yuseong-gu, Daejeon, 34122 Republic of Korea

**Keywords:** Industrial microbiology, Applied microbiology

## Abstract

Microbial lysates, rich in protein and essential nutrients, demonstrate remarkable capabilities in forming gels and stable foams when heated and whisked, similar to liquid eggs. These characteristics make them an excellent alternative to animal-derived liquid eggs, contributing to sustainable food production and consumption while maintaining high nutritional value. Their versatility positions microbial lysates as promising ingredients in culinary applications, offering a sustainable and nutritious substitute.

Eggs are a valuable and affordable source of essential nutrients, including amino acids, vitamins and essential fatty acids^1^. Liquid eggs, in particular, play a crucial role in various cuisines due to their unique physicochemical properties like viscosity, gelation, foaming, and emulsification^2,3^. Recently, there has been growing interest in egg alternatives made from non-animal sources to address economic, sustainability, and ethical concerns associated with traditional egg production from poultry farming^4,5^ (Supplementary Note 1). Given that microbial products are considered promising sources of nutritious and sustainable foods^5–8^, single-cell proteins and recombinant egg proteins produced by engineered microorganisms, as well as plant-derived proteins, are increasingly being used as substitutes for egg products like hard-boiled eggs, egg-based sauces, dressings and bakery items^4,5^.

In this study, we investigated the potential of using microbial biomass, which is high in protein content (Supplementary Tables 1-3), as a substitute for liquid eggs, since liquid egg substitutes offer wider culinary applications than hard-boiled egg substitutes. We hypothesized that heating concentrated microbial biomass (i.e., microbial cell pellets) would lead to gel formation. However, the cell pellet of the probiotic Gram-negative bacterium *Escherichia coli* Nissle 1917 (refs. ^9,10^) resulted in fluid after 10 min of heating at 100 °C (Fig. 1a). Since entanglement of adjacent heat-denatured proteins is responsible for the heat-induced gelation of liquid eggs, the cell walls and the inner/outer cell membranes of bacteria were suspected to function as microscopic egg shells that confine the range of gelation within the cytoplasm (Fig. 1b) and make the heat-treated pellet a fluidic suspension of microscopic boiled eggs. Therefore, drawing a parallel to cooking liquid eggs, we investigated the heating of *E. coli* cell lysate, which is devoid of intact cell walls and membranes, to facilitate gelation on a macroscopic scale (Fig. 1b). Indeed, the *E. coli* cell lysate solidified after 10 min of heating at 100 °C (Fig. 1a), thus supporting our hypothesis.

**Fig. 1:**
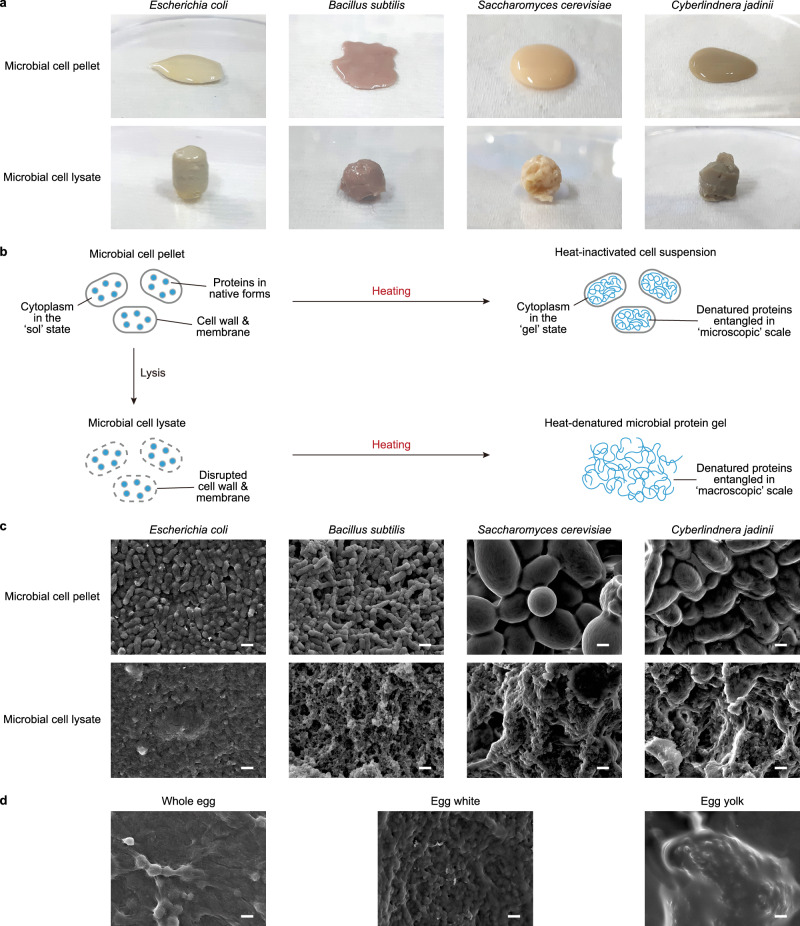
Macroscopic appearance and microscopic structures of the heat-treated microbial cell pellets and lysates. **a** Appearance of the heat-treated (100 °C for 10 min) cell pellet and lysate samples of *E. coli*, *B. subtilis*, *S. cerevisiae* and *C. jadinii*. **b** A schematic describing hypothetical mechanisms for the heat-induced transformation of microbial cell pellets and lysates into fluid and gel forms, respectively. **c** Scanning electron micrographs of the heat-treated (100 °C for 10 min) and freeze-dried cell pellet and lysate samples of *E. coli*, *B. subtilis*, *S. cerevisiae,* and *C. jadinii*. **d** Scanning electron micrographs of the heat-treated (100 °C for 10 min) and freeze-dried samples of whole egg, egg white and egg yolk. Scale bars represent 1 µm.

Similarly, the cell lysate of *Bacillus subtilis*, a model Gram-positive bacterium long consumed as part of traditional fermented foods (e.g., cheonggukjang and natto), also solidified after heat treatment (Fig. 1a). The cell lysate of *Saccharomyces cerevisiae*, representing edible eukaryotic microorganisms, also turned into a gel following heat treatment. However, macroscopic cavities formed within the gel due to the heat-induced release and expansion of CO_2_ actively produced by *S. cerevisiae* (Fig. 1a). The evolution of CO_2_ and formation of cavities were less pronounced when the cell lysate of *Cyberlindnera jadinii* (formerly known as *Candida utilis*), a Crabtree-negative yeast species extensively studied for single-cell protein production^11^, was subjected to heat treatment to create a microbial gel (Fig. 1a).

To further investigate our proposed mechanism (Fig. 1b) for the heat-induced transformation of microbial cell pellets and lysates into fluid and gel forms, respectively, the heat-treated microbial cell pellets and lysates were freeze-dried and their microscopic structures were examined by scanning electron microscopy. Consistent with our hypothesis, the heat-treated microbial cell pellets displayed intact cell shapes (Fig. 1c), resembling a cluster of boiled eggs. In contrast, the surface of the heat-treated microbial cell lysates revealed microscopic structures lacking clear boundaries (Fig. 1c). These structures are similar to those of boiled whole egg (a mixture of egg white and yolk) and boiled egg white (Fig. 1d), supporting heat-induced entanglement of proteins beyond the dimension of microbial cells.

Next, we assessed the mechanical properties of heat-treated microbial cell lysates and compared them with those of heat-treated egg white, egg yolk, and whole egg through compression tests (Supplementary Figs. 1–13 and Supplementary Note 2), which is one of the most popular and well-established methods for assessing the mechanical properties of food materials^12–16^. The apparent Young’s moduli of the heat-treated *E. coli* and *C. jadinii* lysates, measured at 1 mm deformation (19.3 ± 2.7 kPa and 15.9 ± 5.0 kPa, respectively) were similar to that (16.6 ± 0.9 kPa) of diluted [60% (*w/w*)] whole egg (Table 1, Supplementary Figs. 1, 3 and 5). Also, the apparent Young’s modulus of the heat-treated *B. subtilis* lysate was 40.2 ± 13.4 kPa, comparable to those of undiluted whole egg and egg white, which were 37.1 ± 6.7 kPa and 56.6 ± 6.0 kPa, respectively (Table 1, Supplementary Figs. 2, 4 and 9).

**Table 1:** Mechanical properties of the heat-treated samples of whole egg, egg white, egg yolk and various microbial lysates

Heat-treated sample	Apparent Young’s modulus (kPa)	Fracture force (N)	Fracture deformation (mm)	Fracture work (mJ)
*E. coli* lysate	19.3 ± 2.7	0.882 ± 0.070	5.02 ± 0.22	1.829 ± 0.184
*B. subtilis* lysate	40.2 ± 13.4	0.271 ± 0.084	1.38 ± 0.31	0.211 ± 0.101
*C. jadinii* lysate	15.9 ± 5.0	0.300 ± 0.019	3.19 ± 0.20	0.515 ± 0.089
Whole egg (100%)	56.6 ± 6.0	ND	ND	ND
Whole egg (60%)	16.6 ± 0.9	3.650 ± 0.299	6.89 ± 0.27	6.962 ± 0.640
Whole egg (40%)	4.2 ± 0.3	0.836 ± 0.011	5.87 ± 0.23	1.442 ± 0.073
Egg white	37.1 ± 6.7	8.396 ± 0.462	6.75 ± 0.18	14.825 ± 0.729
Egg yolk	125.8 ± 48.0	27.085 ± 0.033	8.11 ± 0.26	62.164 ± 1.625
*E. coli* lysate + heat-inactivated transglutaminase	28.9 ± 2.8	0.699 ± 0.054	4.23 ± 0.16	1.441 ± 0.089
*E. coli* lysate + transglutaminase	31.8 ± 2.4	0.940 ± 0.046	4.57 ± 0.15	1.971 ± 0.148

Values and errors represent mean values and standard deviations of triplicated experimental data (*n* = 3).

*ND* not detected.

Then, the fracture properties such as fracture force, fracture deformation, and fracture work were examined. The heat-treated *E. coli* lysate showed similar properties to those of diluted [40% (*w/w*)] whole egg (Table 1, Supplementary Figs. 1 and 6). On the other hand, the heat-treated *B. subtilis* and *C. jadinii* lysates exhibited significantly lower values (Table 1). The rougher microstructures observed on the cleaved surfaces of the heat-treated *B. subtilis* and *C. jadinii* lysates (Fig. 1c) suggest that the debris of the thicker cell walls may have interfered with the entanglement of microbial proteins in the lysates, leading to the formation of less cohesive gel structures. Nonetheless, the observed mechanical properties indicate that microbial cell lysates are promising substitutes for liquid eggs.

The mechanical properties of heat-treated microbial cell lysates can also be altered by pre-treating them with chemicals or enzymes. For instance, when commercial *Streptomyces mobaraensis* transglutaminase powder, which contains only 0.02 g of microbial protein per 1 g of maltodextrin and is commonly used in food processing to catalyze protein cross-linking^17^, was supplemented to *E. coli* cell lysate at a final concentration of 1% (*w/w*) after heat inactivation, the apparent Young’s modulus increased from 19.3 ± 2.7 kPa to 28.9 ± 2.8 kPa (Table 1, Supplementary Figs. 1 and 7). Also, the fracture force, deformation, and work decreased from 0.882 ± 0.070 N, 5.02 ± 0.22 mm and 1.829 ± 0.184 mJ to 0.699 ± 0.054 N, 4.23 ± 0.16 mm and 1.441 ± 0.089 mJ, respectively. When catalytically active transglutaminase powder was added and incubated at room temperature for 8 h before heat treatment, the fracture force and fracture work values notably increased to 0.940 ± 0.046 N and 1.971 ± 0.148 mJ, respectively, whereas the changes in the apparent Young’s modulus and fracture deformation were not significant (Table 1, Supplementary Figs. 7 and 8).

Stable foam, which is particularly important for baking applications, can also be produced from microbial lysate. A dense foam created by vigorously vortexing *E. coli* lysate remained stable even after 4.5 h, similar to the foam generated from egg white (Supplementary Fig. 12). Furthermore, a microbial meringue batter made by beating *E. coli* lysate with sucrose can be used to bake meringue cookies that resemble meringue cookies made from egg white (Supplementary Fig. 13).

In this study, we demonstrated the potential of microbial lysates as a sustainable alternative to liquid eggs. Firstly, we showed that cell lysates from various edible microorganisms can form gels upon heating, and that the mechanical properties of these gels can be modulated by using different microbial species and supplementing chemicals or enzymes to the lysates. Additionally, we successfully generated stable foam from microbial lysate and used it to bake microbial meringue cookies, a process that heavily relies on the generation of dense and stable foam. Further advancements in microbial strain engineering, development of processing methods, and formulation of recipes to enhance flavor and streamline the lysis of microbial biomass will expedite the adoption of microbial lysates as a sustainable and healthy substitute for liquid eggs.

## Methods

### Microbial strains, plasmids and culture media

Microbial strains and plasmids used in this study are summarized in Supplementary Table 4. The composition of MR medium and modified YM medium (20 g L^−1^ d-glucose, 3 g L^−1^ yeast extract, 3 g L^−1^ malt extract, 5 g L^−1^ peptone) used for flask culture of microbial strains are described in Supplementary Tables 5 and 6.

### Flask culture of microbial strains

For flask culture of *E. coli*, 5 mL MR medium in a 50-mL conical tube was inoculated and incubated overnight at 37 °C with 200 rpm of rotary shaking. Then, 500 µL of the seed culture was transferred to 50 mL MR medium in a 300-mL baffled flask and incubated at 30 °C for 16 h with 200 rpm of rotary shaking. The resulting cell biomass was harvested by centrifugation (at 16,100 × *g* for 2 min or at 2090 × *g* for 10 min) and stored at −80 °C until use.

For flask culture of *B. subtilis*, 5 mL MR medium in a 50-mL conical tube was inoculated and incubated at 37 °C for 24 h with 200 rpm of rotary shaking. Then, 500 µL of the seed culture was transferred to 50 mL MR medium in a 300-mL baffled flask and incubated at 30 °C and 200 rpm. The resulting cell biomass was harvested by centrifugation (at 16,100 × *g* for 2 min or at 2,090 × *g* for 10 min) before the color of the culture turned into pink (i.e., less than 24 h of incubation) and stored at −80 °C until use.

For flask culture of *S. cerevisiae*, 5 mL modified YM medium in a 50-mL conical tube was inoculated and incubated at 30 °C for 24 h with 200 rpm of rotary shaking. Then, 500 µL of the seed culture was transferred to 50 mL modified YM medium in a 300-mL baffled flask and incubated at 30 °C for 16 h with 200 rpm of rotary shaking. The resulting cell biomass was harvested by centrifugation (at 16,100 × *g* for 2 min or at 2090 × *g* for 10 min), washed twice with deionized water to minimize dissolved CO_2_ level and stored at −80 °C until use.

For flask culture of *C. jadinii*, 5 mL MR medium in a 50-mL conical tube was inoculated and incubated overnight at 30 °C with 200 rpm of rotary shaking. Then, 1 mL of the seed culture was transferred to 100 mL MR medium in a 300-mL baffled flask and incubated at 30 °C for 20 h with 200 rpm of rotary shaking. The resulting cell biomass was harvested by centrifugation (at 16,100 × *g* for 2 min or at 2090 × *g* for 10 min), washed twice with deionized water to minimize dissolved CO_2_ level and stored at −80 °C until use.

### Preparation of cell lysates

The microbial cell lysates were prepared by sonicating microbial cell pellets in ice water. For lysis of *S. cerevisiae* and *C. jadinii* cells, deionized water equivalent to 10 and 5% (*w/w*) of the pellet mass (e.g., 100 and 50 mg of water for 1 g of pellet) was supplemented to facilitate the sonication process, respectively. Vibra-Cell VCX 750 (Sonics & Materials, Inc., USA) equipped with an extender probe having a diameter of 13 mm was used to disrupt the microbial cell pellets in a 50-mL conical tube for 20 min (for *E. coli* and *B. subtilis*) or 40 min (for *S. cerevisiae* and *C. jadinii*) of on-time with an amplitude of 35%. For samples in a 1.5-mL microcentrifuge tube, Vibra-Cell VCX 600 (Sonics & Materials, Inc., USA) equipped with a tapered microtip having a diameter of 3 mm was used with an amplitude of 30%. To minimize the rise of temperature during sonication, 9 s of pause time was scheduled after every 1 s of pulse.

### Transglutaminase treatment

For transglutaminase treatment of microbial cell lysates, 0.2 g of commercial *Streptomyces mobaraensis* transglutaminase powder consisting only of maltodextrin and 19.6 mg g^−1^ of protein (Moo Gloo TI transglutaminase, Modernist Pantry, USA) was added to 20 g of microbial cell lysate. Heat-inactivated transglutaminase prepared by incubating the powder at 70 °C for 4 h was used instead for preparation of control samples. The mixture was vigorously vortexed to facilitate dissolution of the commercial transglutaminase powder to the microbial cell lysate and spun down to eliminate bubbles. Then, the mixture was incubated at room temperature until heat treatment.

### Preparation of the heat-treated samples

To prepare the heat-treated samples of unlysed microbial pellets, microbial lysates or liquid eggs, the raw samples were transferred to 1.5- or 2-mL microcentrifuge tubes, briefly spun down to remove air bubbles inside the sample and incubated at 100 °C for 10 or 60 min (for scanning electron microscopy or texture profile analysis, respectively) using a heat block. For easier displacement of the gel samples, the bottoms of the 2-mL microcentrifuge tubes were removed using a PVC tube cutter, and the samples were pushed to the bottom using a plunger or pulled down by gravity. The resulting samples for texture profile analysis were stored at 4 °C until use.

### Scanning electron microscopy

The microstructure of the heat-treated samples was observed by scanning electron microscopy. To prepare the specimens for scanning electron microscopy, the heat-treated samples were freeze-dried, and particles derived from them were attached to the stage using a carbon tape. Then, the surface of the specimens was coated with osmium with a thickness of ~4 nm using HPC-1SW osmium coater (Shinkuu, Japan) and examined using JSM-IT800 field emission scanning electron microscope (JEOL Ltd., Japan) at KAIST Analysis center for Research Advancement (KARA).

### Texture profile analysis

The texture profile of the heat-treated samples was assessed by compression test. To prepare gel samples for compression test, the heat-treated samples stored at 4 °C were warmed at room temperature and both ends were trimmed to generate cylinders with a length and a diameter of 10 and 8.5 mm. Compression test was conducted using the TXA™ Multi-axis Micro-Texture Analyzer (TXA™ -Precision model, YEONJIN S-TECH, Republic of Korea) equipped with a 1-, 3- or 6-kgf load cell and a cylindrical aluminum probe with a diameter of 35 mm at room temperature. The gel samples were compressed at 2 mm/s with a data sampling frequency of 300 point/s until the probe reached the 2 mm point from the bottom. Submillimetre-scale shifts in the compression start points, which originate from the submillimetre-scale deviations in the trimmed sample length, were corrected by monitoring the measured force values.

The apparent Young’s moduli of the heat-treated samples were calculated by dividing the force applied at 1 mm deformation by the strain at 1 mm deformation (= 1 mm/10 mm = 0.1) and the initial cross-sectional area of the heat-treated sample (=*π* × 8.5 mm × 8.5 mm/4 = 56.745 mm^2^). For example, if 1 N is applied at 1 mm deformation, the apparent Young’s modulus is calculated to be 176.2 kPa (=1 N/0.1/56.745 mm^2^ = 0.1762 N mm^−2^).

The fracture force and deformation were determined at the first prominent peak or its preceding prominent shoulder observed in a force-deformation graph. To calculate the fracture work, the amount of work done between two neighboring sampling points was calculated by multiplying the deformation distance (=0.01 mm) and the mean force exerted over the distance (e.g., 1.2 N for a sampling interval with the initial and final force values of 1.1 N and 1.3 N). Then, the multiplication products from all sampling intervals between the compression start point and the fracture point were summed up to calculate the fracture work.

### Preparation of whipped microbial cell lysate

Five grams of the cell lysate in a 50-mL conical tube was vigorously vortexed for 5 min to generate foams from the microbial cell lysates. The resulting product was incubated at room temperature with or without spin-down to observe the stability of the generated foams.

### Baking microbial meringue cookies

Microbial meringue cookies were baked following a typical recipe for meringue cookies. To beat meringue from microbial cell lysate, 5 g of microbial cell lysate in 50-mL conical tube was first vigorously vortexed to generate enough foam. While whipping the microbial lysate with a pair of single-use loops, 5 g of sucrose (Junsei, Japan) were added in three portions. The next portion of sucrose was added after previously added portion of sucrose was completely dissolved into the microbial lysate. The microbial meringue was whipped until it became thick enough and did not fall out of the tube when turned over. Then, the microbial meringue was transferred to a piping bag, piped onto a single-use plastic plate and baked for 1.5 h in a forced convection oven (OF-12GW, Lab Companion, Republic of Korea) preheated to 130 °C. The resulting microbial meringue cookies were cooled at room temperature.

### Crude component analysis of freeze-dried microbial biomass

Water content of a freeze-dried microbial biomass sample was determined using an ambient pressure heat-drying method. Briefly, ~2 g of sample was weighed, dried at 135 °C for 2 h using a forced convection oven (JSOF-150, JSR Co., Republic of Korea), cooled in a desiccator for more than 30 min and weighed again. The water content was calculated by dividing the weight loss upon the ambient pressure heat-drying with the original weight.

Crude protein content was determined by multiplying a nitrogen-to-protein conversion factor of 6.25 to the nitrogen content of a freeze-dried sample^18^. The nitrogen content of a sample was determined by the Kjeldahl method, as described in the AOAC 976.05 (ref. ^19^). Briefly, selenium catalyst (Kjeltabs S-3.5, FOSS TECATOR, Höganäs, Sweden) and 12 mL of sulfuric acid were supplemented to ~0.5 g of the freeze-dried sample and incubated at 420 °C for 1 h, and the nitrogen content was analyzed using Kjeltec auto 2300 Analyzer (FOSS TECATOR, Höganäs, Sweden).

Crude fat content was determined through Soxhlet extraction using ST 243 Soxtec^TM^ manual solvent extraction system (FOSS TECATOR, Höganäs, Sweden). Briefly, a cellulose thimble was filled with 1 g of the freeze-dried sample, plugged with cotton and installed to ST 243 Soxtec^TM^. Also, an extraction cup was filled with 80 mL of ethyl ether after measuring the empty weight and installed to ST 243 Soxtec^TM^. Next, the fats in the freeze-dried sample were extracted through following steps: 20 min of boiling at 110 °C; 40 min of rinsing; 20 min of recovery; 20 min of drying; further evaporation of residual ethyl ether in a fume hood. Then, the extraction cup was additionally dried up at 110 °C for 1 h, cooled in vacuum for 30 min and weighed to measure the weight increase. The increase in the weight of the extraction cup per unit mass of the freeze-dried sample put into the cellulose thimble was considered the crude fat content of the sample.

To determine the ash content, ~2 g of the freeze-dried sample contained in a crucible was pre-combusted using an electric stove and additionally combusted at 600 °C for 2 h in an electric furnace (CT-DMF2, Coretech Co., Republic of Korea). The combusted sample was cooled in a desiccator for 40 min, and the resulting ash mass per unit mass of the freeze-dried sample was considered the ash content.

The proportion of the remainder, excluding the water, crude protein, crude fat and ash contents, was considered the crude carbohydrate content.

### Amino acid composition analysis of freeze-dried microbial biomass

Amino acid composition of the freeze-dried microbial biomass was analyzed using a modified AOAC method^20^ based on ion-exchange chromatography (IEC) and post-column derivatization (PCD) using ninhydrin. To quantify acid-stable, non-sulfur amino acids, 0.2 g of the freeze-dried sample and 10 mL of 6 N HCl aqueous solution were added into a hydrolysis tube purged with N_2_ and incubated at 110 °C for 24 h to hydrolyze proteins into amino acids. The resulting sample was concentrated using a vacuum evaporator, diluted with 0.2 M sodium citrate buffer to a final volume of 50 mL and filtered using a 0.2-μm cellulose acetate syringe filter for subsequent analysis based on IEC and PCD. Performic acid oxidation method was used to prepare samples for quantifying methionine and cysteine, which contain sulfur.

Twenty microlitres of the pre-treated samples were injected into an amino acid analyzer (L-8900, Hitachi, Japan) equipped with an ion exchange column (#2622PH column, 4.6 × 60 mm) and a UV/VIS detector. PH-SET KANTO buffer set (KANTO, Japan) was used as mobile phase at a flow rate of 0.35 mL min^−1^ and ninhydrin reagent kit for Hitachi (WAKO, Japan) was supplied at a flow rate of 0.30 mL min^−1^. The absorbance peaks of the derivatized amino acids were detected at wavelength of 440 or 570 nm.

Alkaline hydrolysis method was applied to prepare samples for quantifying tryptophan, which is unstable under acidic conditions. Twenty microlitres of the pre-treated samples were injected to a high-performance liquid chromatography system equipped with isocratic 600 pump and 486 UV/VIS detector (Waters, Massachusetts, United States). CAPCELL PAK C18 (4.6 × 250 mm; PerkinElmer, Massachusetts, United States) was isocratically eluted with a mixture of 8.5 mM sodium acetate solution and methanol (volumetric ratio of 95:5) at 1.0 mL min^−1^. The absorbance of the eluted peaks were detected at wavelength of 280 nm.

### Supplementary information

Supplementary Information

## Data Availability

All source data for figures have been deposited at Figshare (10.6084/m9.figshare.26015500).
