# Author Correction: Microbial lysates repurposed as liquid egg substitutes

**DOI:** 10.1038/s41538-024-00289-4

**Published:** 2024-07-15

**Authors:** Kyeong Rok Choi, Da-Hee Ahn, Seok Yeong Jung, Yu Hyun Lee, Sang Yup Lee

**Affiliations:** 1 Metabolic and Biomolecular Engineering National Research Laboratory, Systems Metabolic Engineering and Systems Healthcare Cross-Generation Collaborative Laboratory, Department of Chemical and Biomolecular Engineering (BK21 four), Korea Advanced Institute of Science and Technology (KAIST), Daejeon, 34141 Republic of Korea; 2 BioProcess Engineering Research Center, KAIST, Daejeon, 34141 Republic of Korea; 3 BioInformatics Research Center, KAIST Institute for the BioCentury, KAIST Institute for Artificial Intelligence, KAIST, Daejeon, 34141 Republic of Korea; 4 Graduate School of Engineering Biology, KAIST, Daejeon, 34141 Republic of Korea; 5 Present Address: Research and Development Center, GS Caltex Corporation, Yuseong-gu, Daejeon, 34122 Republic of Korea

**Keywords:** Industrial microbiology, Applied microbiology

Correction to: *npj Science of Food* 10.1038/s41538-024-00281-y, published online 19 June 2024

In the Original Article, ‘C. jadinii’’ was incorrectly given as ‘C. necator’ in the subsection ‘Preparation of cell lysates’ in the Methods section.

The original article has been corrected.

