# Supplementary material for: Microbial lysates repurposed as liquid egg substitutes
Source: NPJ Sci Food. 2024 Jun 19;8:35. doi: 10.1038/s41538-024-00281-y (PMC11187216; doi:10.1038/s41538-024-00281-y)
Supplement: Supplementary file 1 — Supplementary Information [file 41538_2024_281_MOESM1_ESM.pdf]

Supplementary Information for

Microbial lysates repurposed as liquid egg substitutes

Kyeong Rok Choi[1,2,5], Da-Hee Ahn[1], Seok Yeong Jung[1], Yu Hyun Lee[1,2] and Sang Yup Lee[1,2,3,4,*]

[1]Metabolic and Biomolecular Engineering National Research Laboratory, Systems Metabolic Engineering and Systems Healthcare Cross-Generation Collaborative Laboratory, Department of Chemical and Biomolecular Engineering (BK21 four), Korea Advanced Institute of Science and Technology (KAIST), Daejeon 34141, Republic of Korea.

[2]BioProcess Engineering Research Center, KAIST, Daejeon 34141, Republic of Korea.

[3]BioInformatics Research Center, KAIST Institute for the BioCentury, KAIST Institute for Artificial Intelligence, KAIST, Daejeon 34141, Republic of Korea.

[4]Graduate School of Engineering Biology, KAIST, Daejeon 34141, Republic of Korea.

[5]Current address: Research and Development Center, GS Caltex Corporation, Yuseong-gu, Daejeon 34122, Republic of Korea

*Correspondence should be addressed to Sang Yup Lee.

e-mail: leesy@kaist.ac.kr

Supplementary Figures

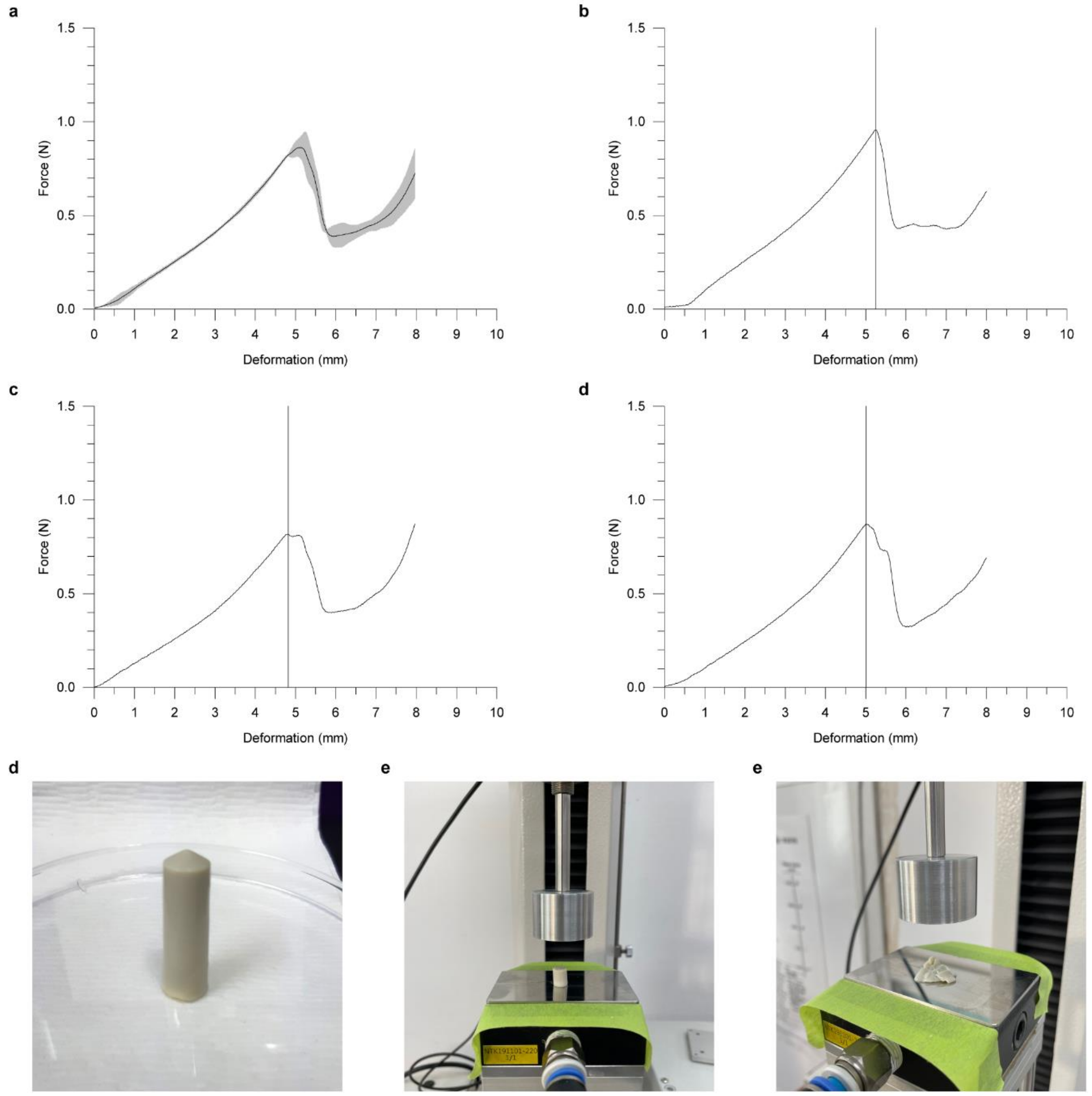

Supplementary Fig. 1 | Compression test of the heat-treated *E. coli* cell lysate. a, Averaged profile of the compression test. Black curve and grey shades represent the mean values and standard deviations of triplicate data (n = 3), respectively. **b–d**, Raw data of the triplicated compression test profiles. Black curves and vertical lines represent measured data and fracture point, respectively. **e**, Appearance of the heat-treated *E. coli* cell lysate sample isolated from the mould. **f**, Appearance of the trimmed specimen before compression test. **g**, Appearance of the specimen after the compression test.

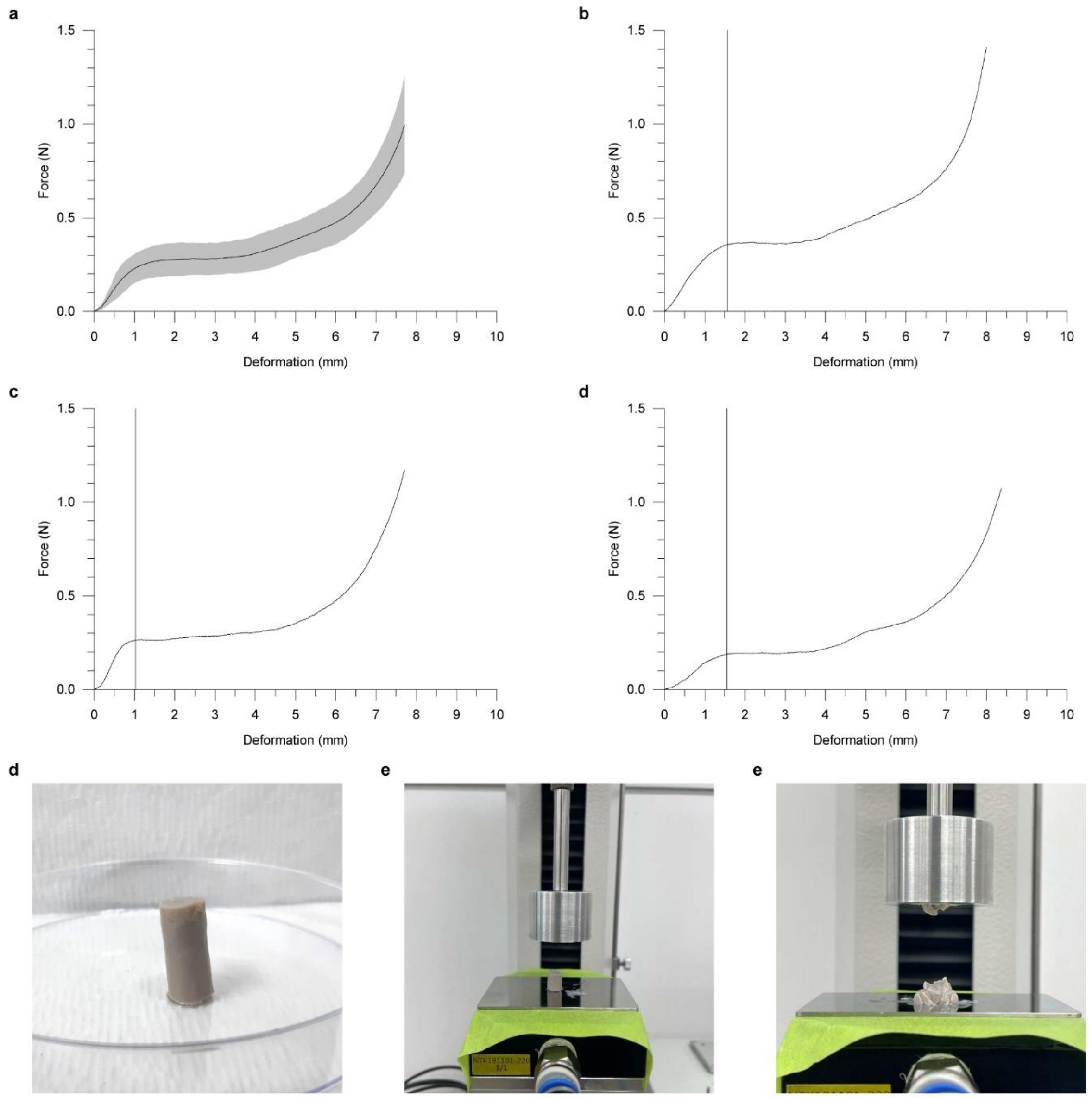

Supplementary Fig. 2 | Compression test of the heat-treated *B. subtilis* cell lysate. a, Averaged profile of the compression test. Black curve and grey shades represent the mean values and standard deviations of triplicate data (n = 3), respectively. **b–d**, Raw data of the triplicated compression test profiles. Black curves and vertical lines represent measured data and fracture point, respectively. **e**, Appearance of the heat-treated *B. subtilis* cell lysate sample isolated from the mould. **f**, Appearance of the trimmed specimen before compression test. **g**, Appearance of the specimen after the compression test.

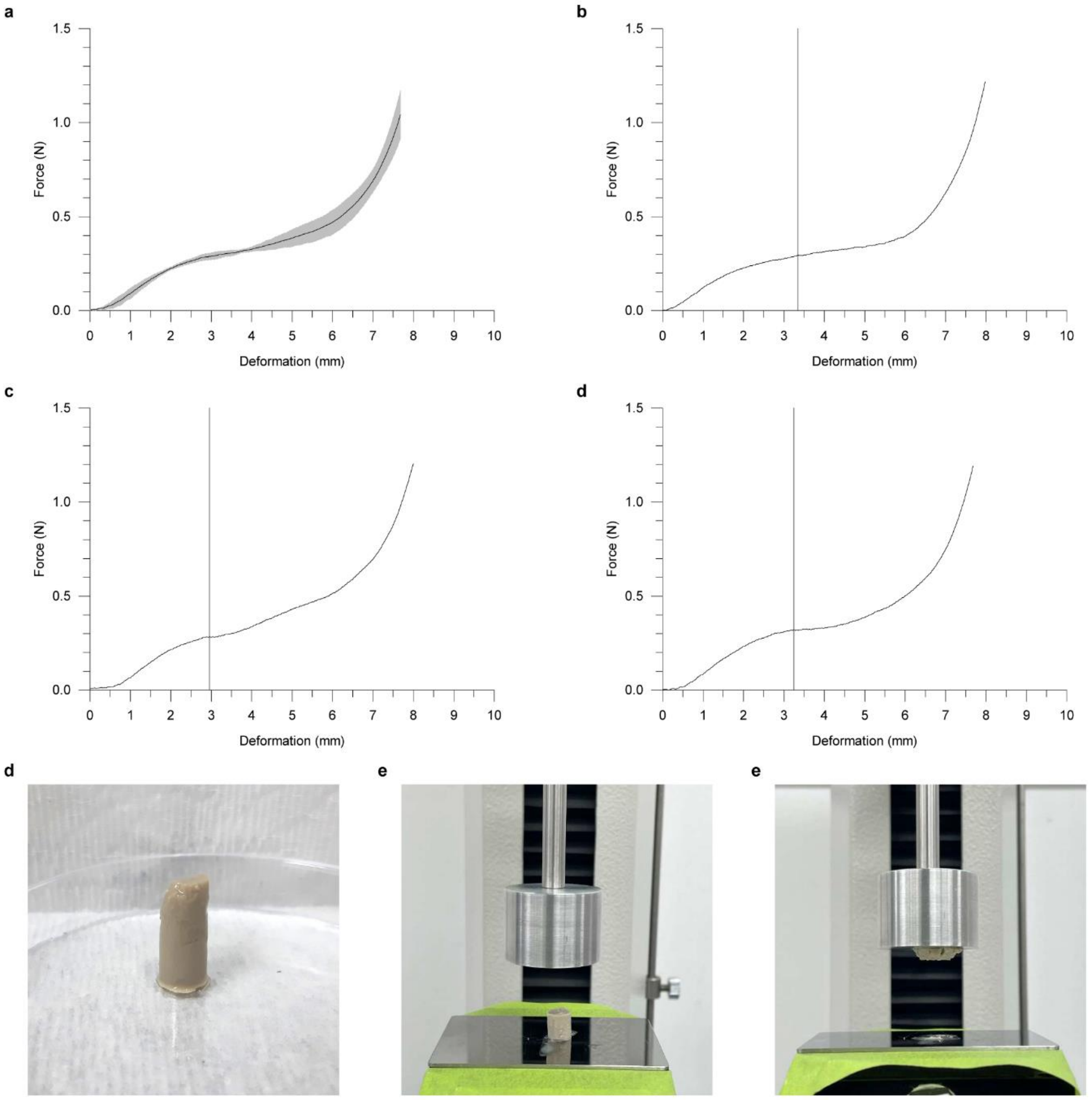

Supplementary Fig. 3 | Compression test of the heat-treated *C. jadinii* cell lysate. a, Averaged profile of the compression test. Black curve and grey shades represent the mean values and standard deviations of triplicate data (n = 3), respectively. **b–d**, Raw data of the triplicated compression test profiles. Black curves and vertical lines represent measured data and fracture point, respectively. **e**, Appearance of the heat-treated *C. jadinii* cell lysate sample isolated from the mould. **f**, Appearance of the trimmed specimen before compression test. **g**, Appearance of the specimen after the compression test.

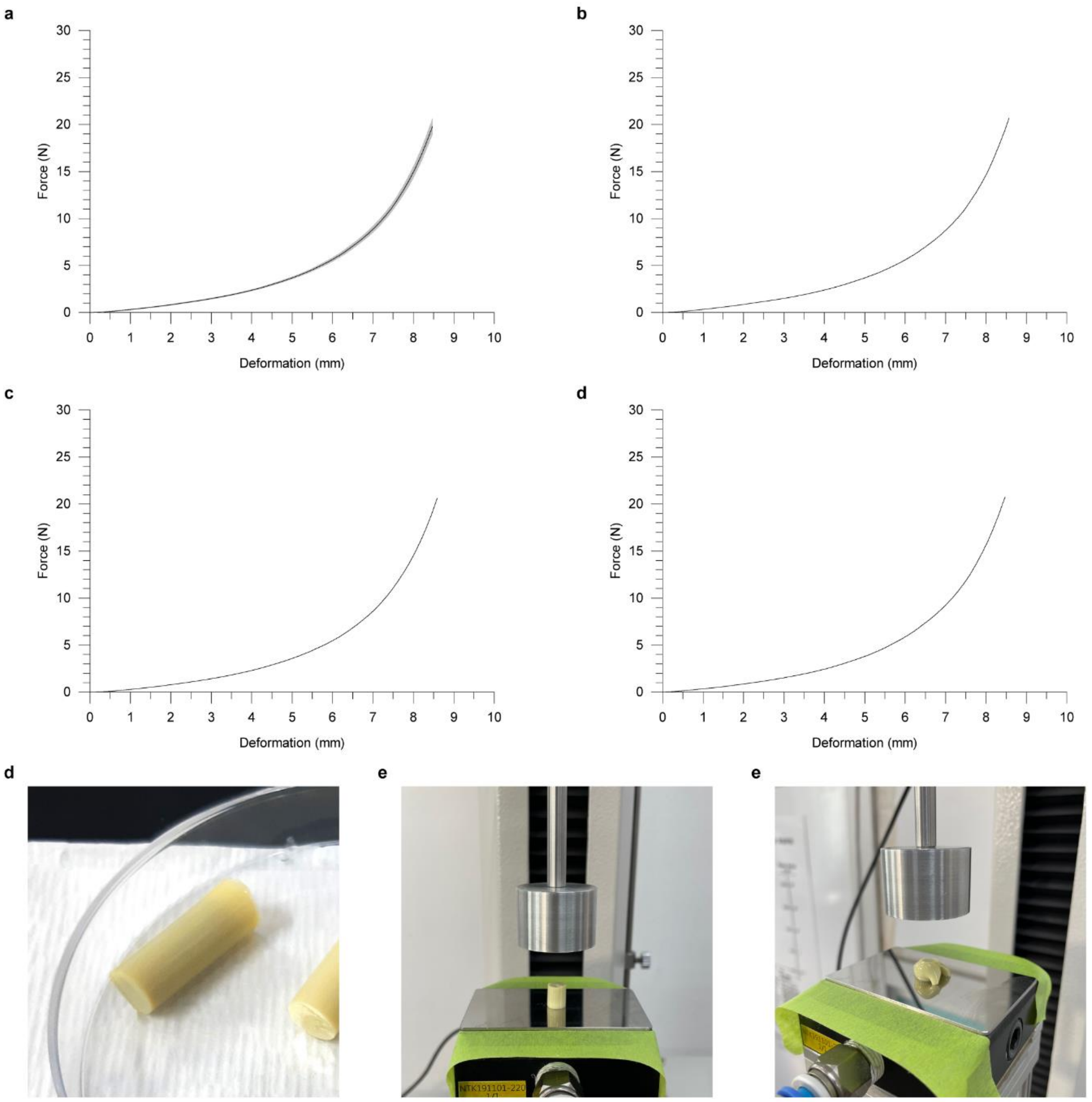

Supplementary Fig. 4 | Compression test of the heat-treated 100% (w/w) whole egg. a, Averaged profile of the compression test. Black curve and grey shades represent the mean values and standard deviations of triplicate data (n = 3), respectively. **b–d**, Raw data of the triplicated compression test profiles. Black curves and vertical lines represent measured data and fracture point, respectively. **e**, Appearance of the heat-treated 100% (w/w) whole egg sample isolated from the mould. **f**, Appearance of the trimmed specimen before compression test. **g**, Appearance of the specimen after the compression test.

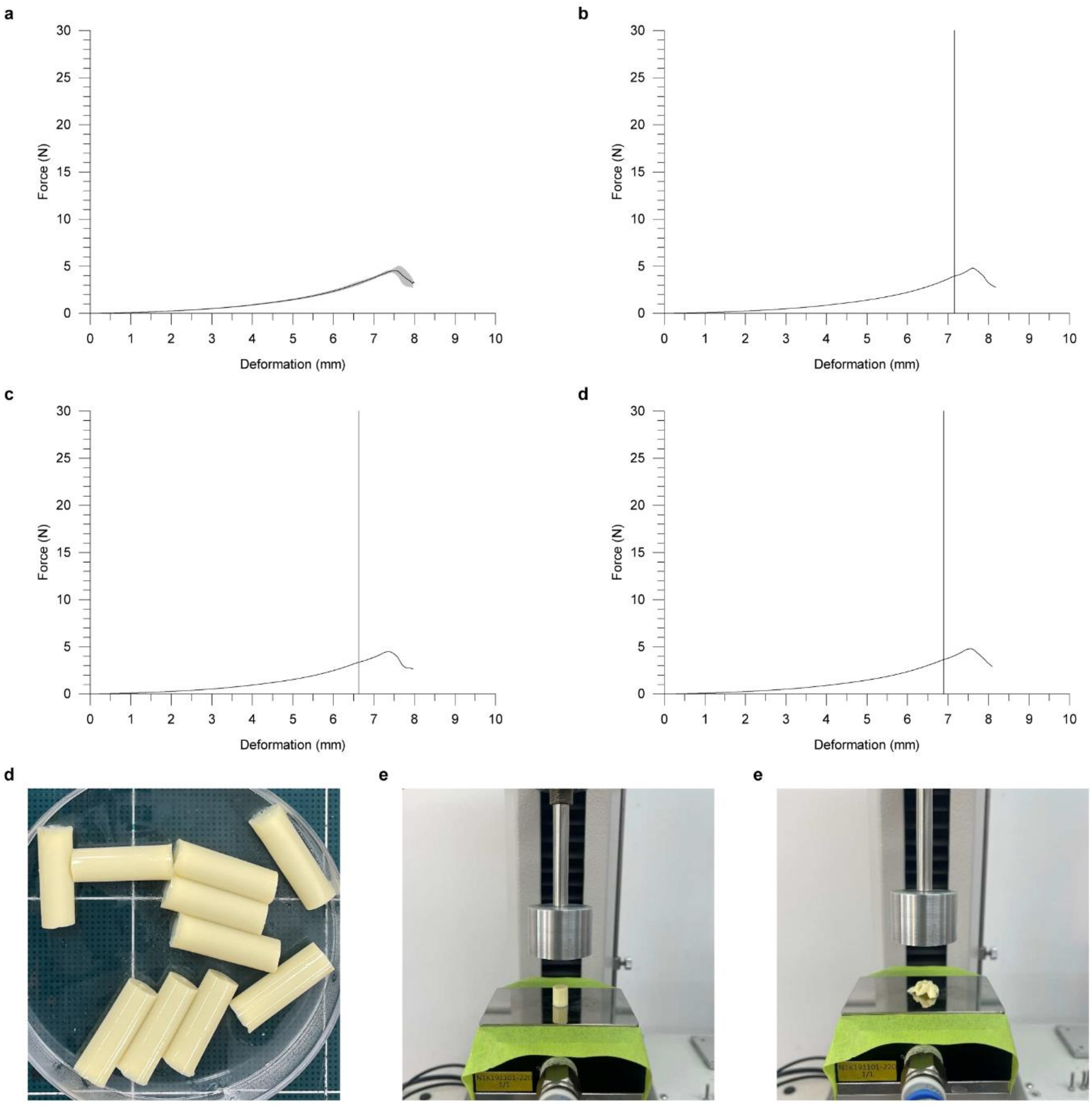

Supplementary Fig. 5 | Compression test of the heat-treated 60% (w/w) whole egg. a, Averaged profile of the compression test. Black curve and grey shades represent the mean values and standard deviations of triplicate data (n = 3), respectively. **b–d**, Raw data of the triplicated compression test profiles. Black curves and vertical lines represent measured data and fracture point, respectively. **e**, Appearance of the heat-treated 60% (w/w) whole egg sample isolated from the mould. **f**, Appearance of the trimmed specimen before compression test. **g**, Appearance of the specimen after the compression test.

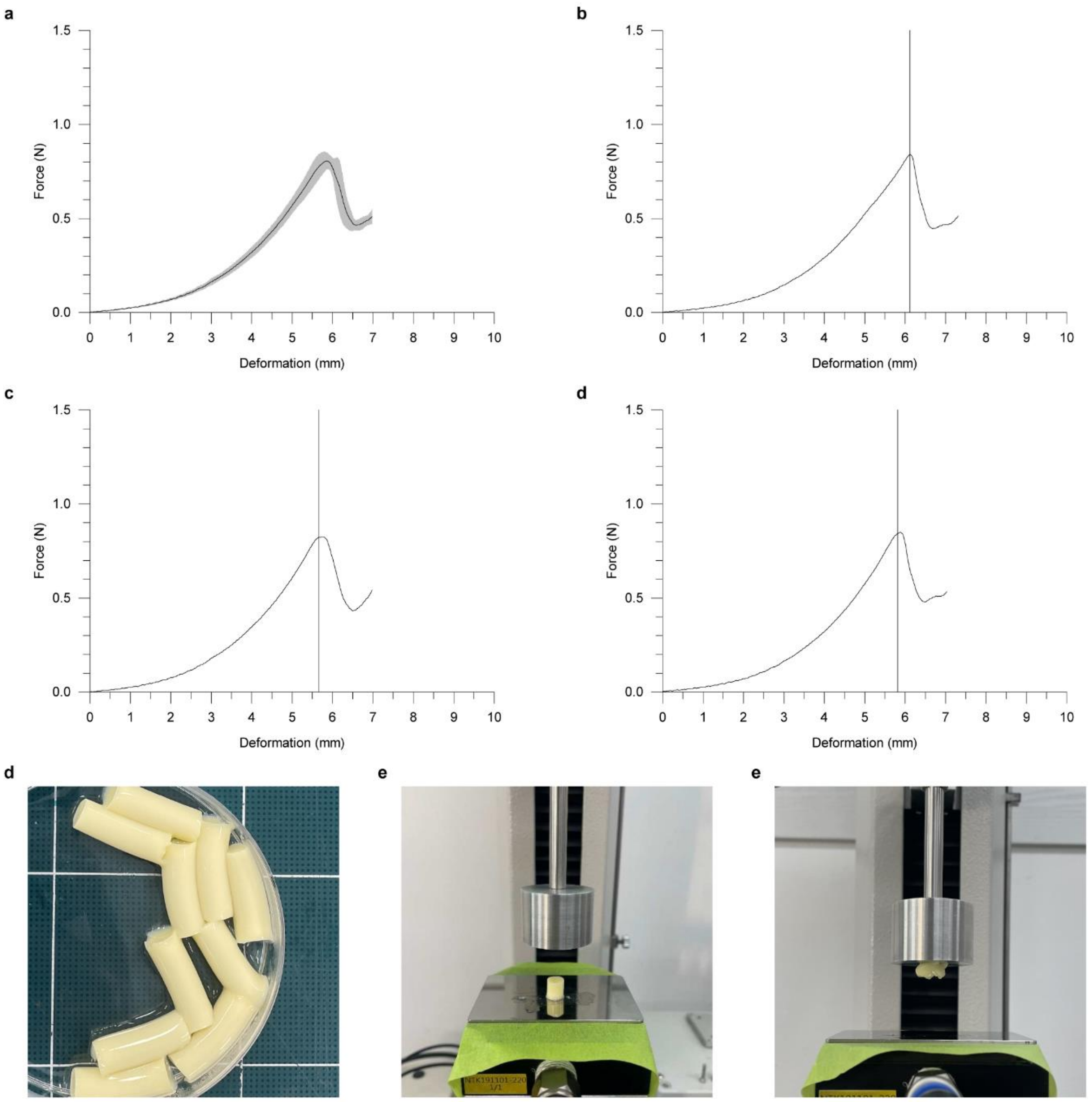

Supplementary Fig. 6 | Compression test of the heat-treated 40% (w/w) whole egg. a, Averaged profile of the compression test. Black curve and grey shades represent the mean values and standard deviations of triplicate data (n = 3), respectively. **b–d**, Raw data of the triplicated compression test profiles. Black curves and vertical lines represent measured data and fracture point, respectively. **e**, Appearance of the heat-treated 40% (w/w) whole egg sample isolated from the mould. **f**, Appearance of the trimmed specimen before compression test. **g**, Appearance of the specimen after the compression test.

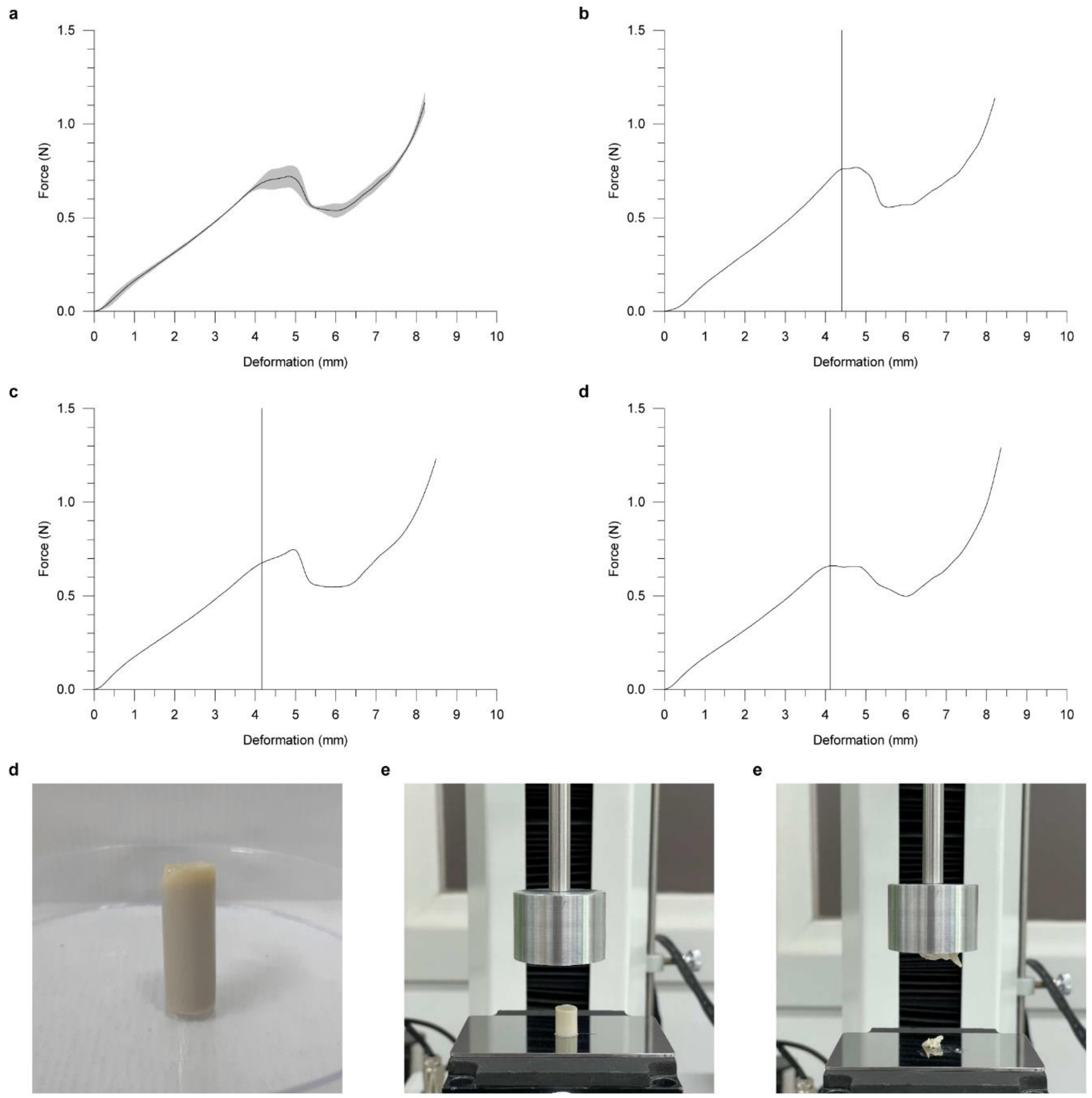

Supplementary Fig. 7 | Compression test of the heat-treated *E. coli* cell lysate supplemented with heat-inactivated transglutaminase powder. a, Averaged profile of the compression test. Black curve and grey shades represent the mean values and standard deviations of triplicate data ($n = 3$), respectively. **b–d**, Raw data of the triplicated compression test profiles. Black curves and vertical lines represent measured data and fracture point, respectively. **e**, Appearance of the heat-treated *E. coli* cell lysate sample that was supplemented with heat-inactivated transglutaminase powder, heat-treated and isolated from the mould. **f**, Appearance of the trimmed specimen before compression test. **g**, Appearance of the specimen after the compression test.

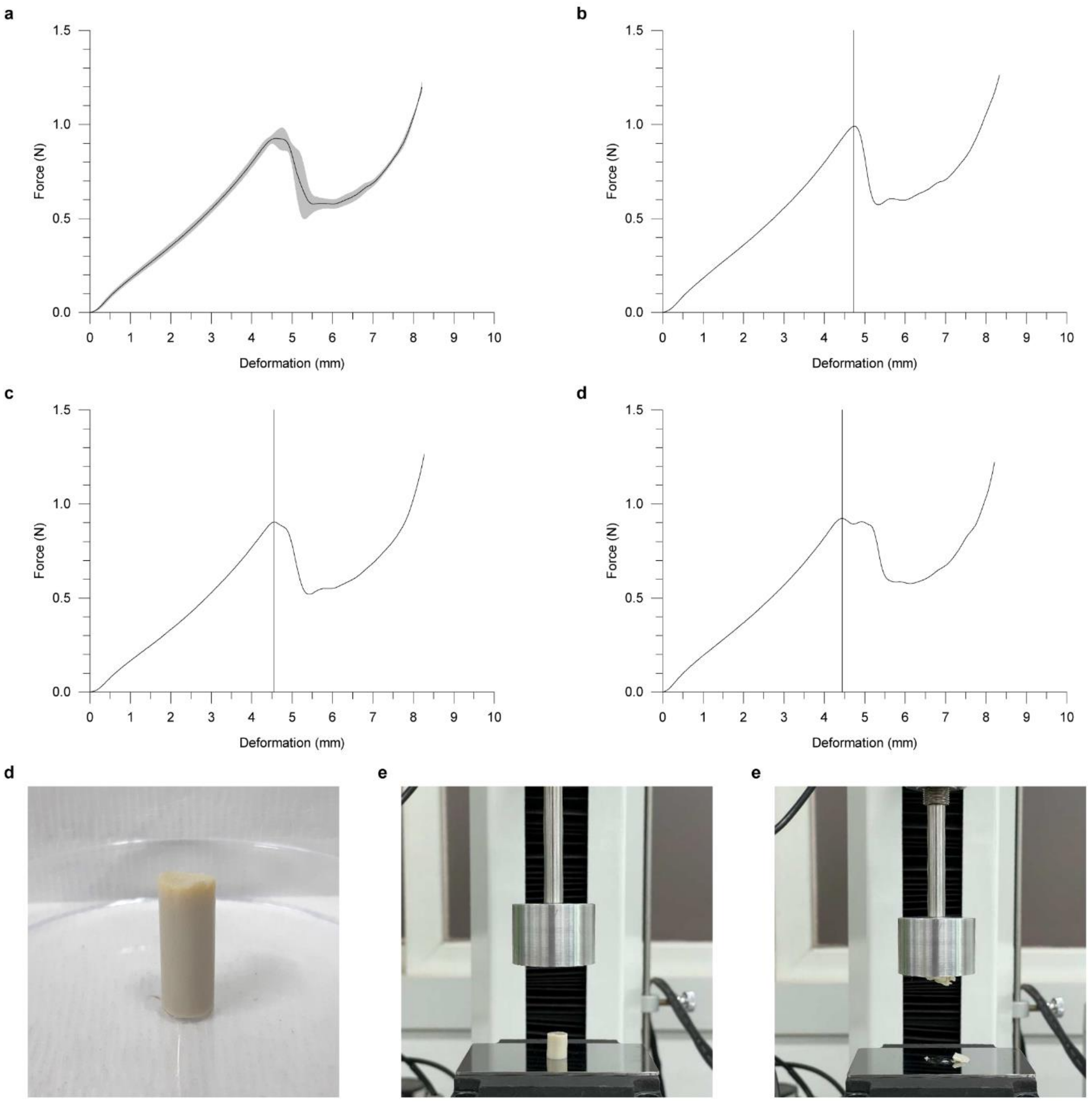

Supplementary Fig. 8 | Compression test of the heat-treated *E. coli* cell lysate supplemented and incubated (8 h) with catalytically active transglutaminase powder. a, Averaged profile of the compression test. Black curve and grey shades represent the mean values and standard deviations of triplicate data (n = 3), respectively. **b–d**, Raw data of the triplicated compression test profiles. Black curves and vertical lines represent measured data and fracture point, respectively. **e**, Appearance of the heat-treated *E. coli* cell lysate sample that was supplemented with catalytically active transglutaminase powder, incubated for 8 h at room temperature, heat-treated and isolated from the mould. **f**, Appearance of the trimmed specimen before compression test. **g**, Appearance of the specimen after the compression test.

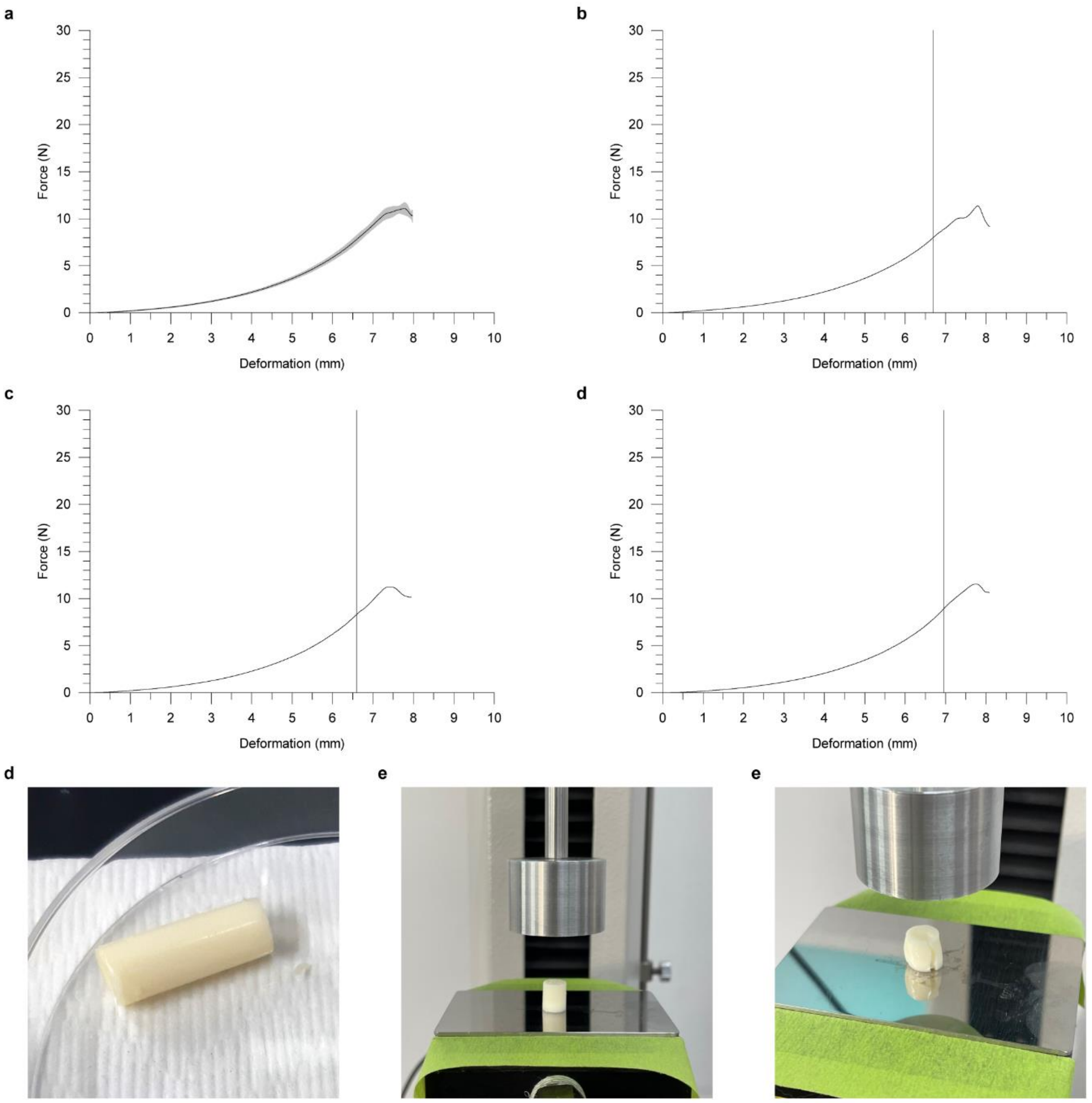

Supplementary Fig. 9 | Compression test of the heat-treated 100% (*w/w*) egg white. a, Averaged profile of the compression test. Black curve and grey shades represent the mean values and standard deviations of triplicate data (n = 3), respectively. **b–d**, Raw data of the triplicated compression test profiles. Black curves and vertical lines represent measured data and fracture point, respectively. **e**, Appearance of the heat-treated 100% (*w/w*) egg white sample isolated from the mould. **f**, Appearance of the trimmed specimen before compression test. **g**, Appearance of the specimen after the compression test.

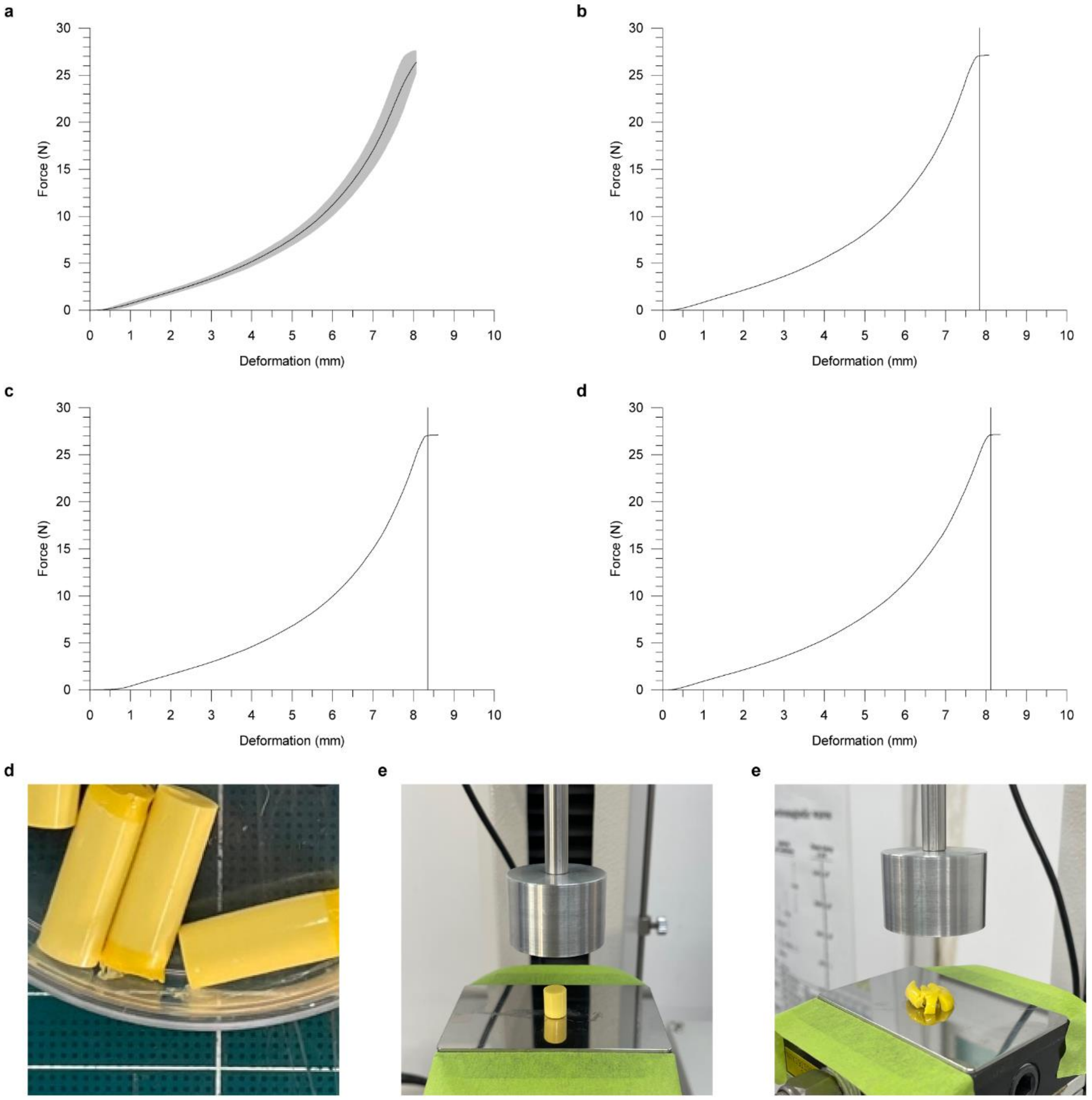

Supplementary Fig. 10 | Compression test of the heat-treated 100% (*w/w*) egg yolk. a, Averaged profile of the compression test. Black curve and grey shades represent the mean values and standard deviations of triplicate data (n = 3), respectively. **b–d**, Raw data of the triplicated compression test profiles. Black curves and vertical lines represent measured data and fracture point, respectively. **e**, Appearance of the heat-treated 100% (*w/w*) egg yolk sample isolated from the mould. **f**, Appearance of the trimmed specimen before compression test. **g**, Appearance of the specimen after the compression test.

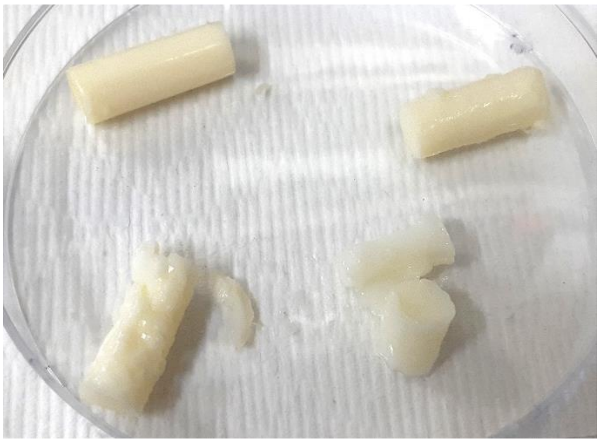

Supplementary Fig. 11 | Appearance of heat-treated samples of diluted or undiluted liquid egg white. Undiluted liquid egg white specimen (upper left) could be isolated from the mould in an intact form after the heat treatment. In contrast, liquid egg whites diluted with deionized water at 60% (*w/w*; upper right), 50% (*w/w*; lower left) and 40% (*w/w*; lower right) attached to the wall of the mould upon the heat treatment and hence intact specimens could not be isolated from the mould.

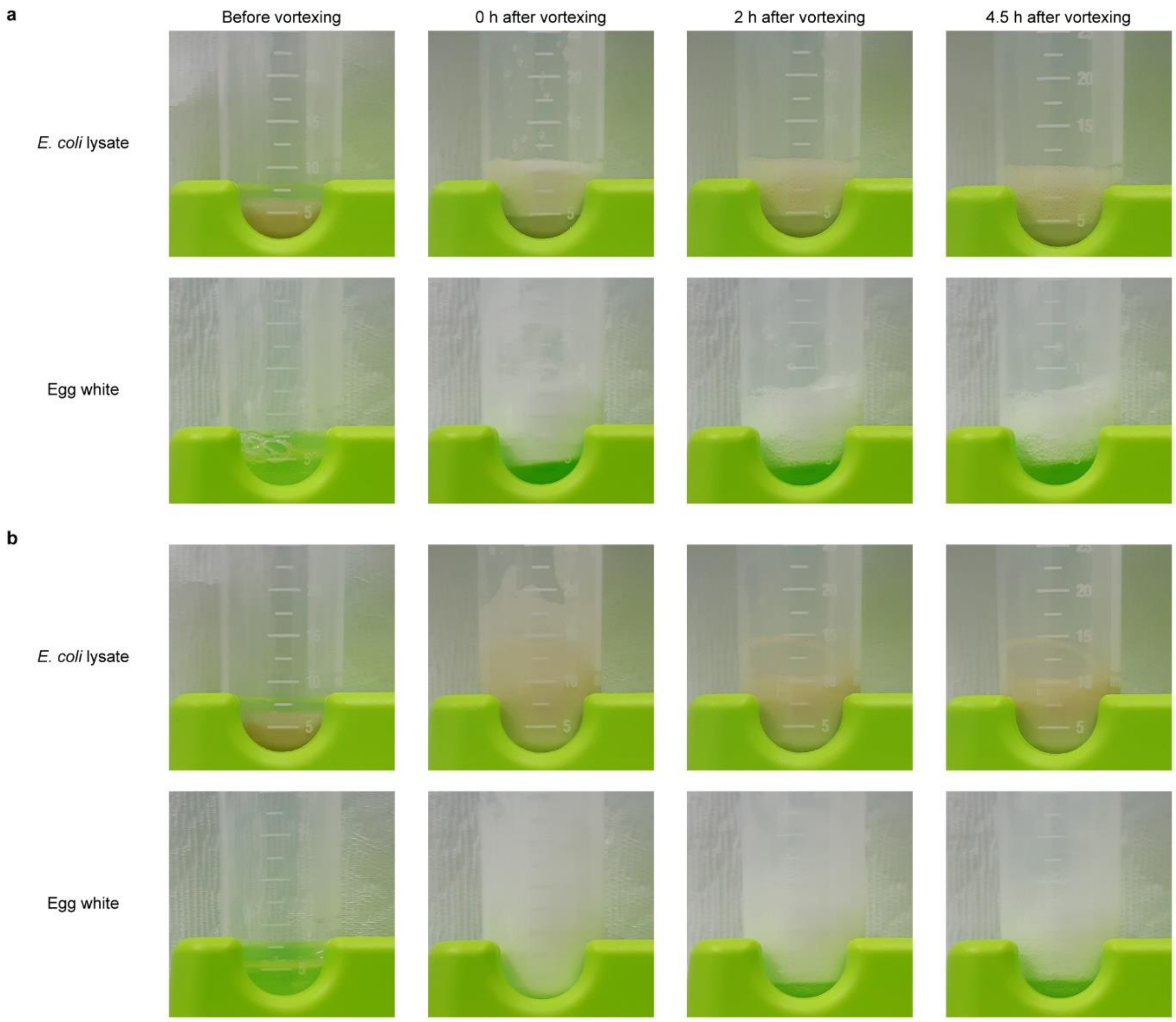

Supplementary Fig. 12 | Stability of foam produced from *E. coli* cell lysate or egg white. Five grams of *E. coli* cell lysate or egg white (before vortexing) was vigorously vortexed to produce foam (0 h after vortexing) and either spinned down (**a**) or not (**b**). The stability of the foam was observed by statically incubating at room temperature for up to 4.5 h.

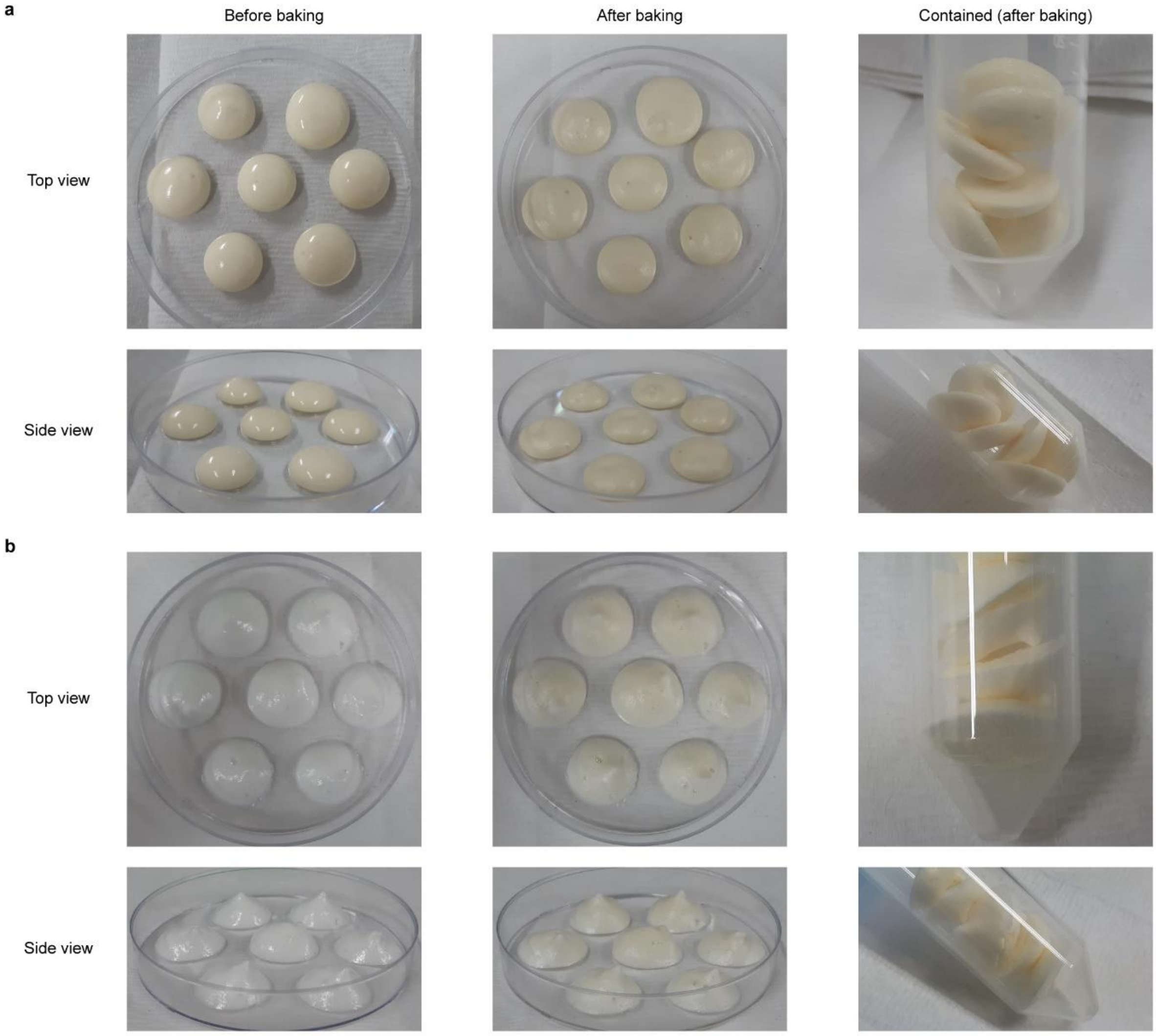

Supplementary Fig. 13 | Meringue cookies baked from *E. coli* cell lysate or egg white. a, Microbial meringue was prepared by beating an equal mass of *E. coli* cell lysate and sucrose, then piped onto a plastic plate (before baking). The piped meringue batter was baked at 130 °C for 1.5 h and then allowed to cool down at room temperature (after baking). The baked microbial meringue cookies were detached from the plastic plate and placed in a 50-mL conical tube [contained (after baking)]. **b**, Conventional meringue was made by beating an equal mass of egg white and sucrose, then piped onto a plastic plate (before baking). The piped meringue batter was baked at 130 °C for 1.5 h and then allowed to cool down at room temperature (after baking). The baked meringue cookies were detached from the plastic plate and placed in a 50-mL conical tube [contained (after baking)].

Supplementary Tables

Supplementary Table 1. Biomass compositions of some representative microbial species[a]

Component	***Escherichia coli* Nissle 1917**	***Bacillus subtilis* 168 Δ*sigF-spoVAF***	***Saccharomyces cerevisiae***	***Cyberlindnera jadinii***
Water	2.83 ± 0.03	2.08 ± 0.01	5.08	-
Crude protein	72.01 ± 0.45	70.29 ± 0.20	40.4	54.8 ± 0.12
Crude fat	1.86 ± 0.18	5.28 ± 0.26	7.61	15.12 ± 0.98
Ash	16.68 ± 0.15	15.55 ± 0.20	5.65	8.10 ± 0.18
Crude carbohydrate	6.61 ± 0.11	6.80 ± 0.67	41.2	2.80± 0.20
Aspartic acid	6.06 ± 0.03	6.35 ± 0.06	4.15	8.00 ± 0.72
Threonine	2.83 ± 0.01	2.56 ± 0.03	1.99	4.10 ± 0.58
Serine	2.20 ± 0.02	1.97 ± 0.02	1.98	3.60 ± 1.02
Glutamic acid	7.02 ± 0.05	9.89 ± 0.11	6.47	15.30 ± 0.66
Proline	1.91 ± 0.03	1.51 ± 0.01	1.65	2.80 ± 0.23
Glycine	3.15 ± 0.02	2.43 ± 0.01	1.93	3.80 ± 1.96
Alanine	4.41 ± 0.01	4.13 ± 0.03	2.32	6.90 ± 0.58
Valine	3.50 ± 0.01	4.21 ± 0.01	2.31	5.50 ± 0.15
Isoleucine	2.70 ± 0.02	2.71 ± 0.03	1.89	4.80 ± 0.99
Leucine	4.78 ± 0.03	9.63 ± 0.07	2.92	7.12 ± 1.64
Tyrosine	1.81 ± 0.08	1.49 ± 0.04	1.13	2.40 ± 1.20
Phenylalanine	2.42 ± 0.01	2.06 ± 0.01	1.75	4.10 ± 0.25
Histidine	1.24 ± 0.01	1.06 ± 0.01	0.91	-
Lysine	4.32 ± 0.01	4.03 ± 0.03	3.28	5.14 ± 0.82
Arginine	3.74 ± 0.04	2.64 ± 0.01	2.03	3.20 ± 1.12
Cysteine	0.71 ± 0.01	0.42 ± 0.02	0.50	-
Methionine	1.66 ± 0.03	1.50 ± 0.05	0.59	1.58 ± 2.10
Tryptophan	0.67 ± 0.03	0.38 ± 0.02	0.54	3.90 ± 0.78
Source	This study	This study	USDA FoodData Central[b]	Ref. [1]

[a]Compositions in g per 100 g dried biomass.
[b]https://fdc.nal.usda.gov/fdc-app.html#/food-details/175043/nutrients

Supplementary Table 2. Raw data for the *Escherichia coli* Nissle 1917 biomass composition presented in Supplementary Table 1[a]

**Component (%)**	**#1**	**#2**	**#3**
Water	2.83	2.80	2.86
Crude protein	71.62	72.50	71.92
Crude fat	2.01	1.66	1.92
Ash	16.85	16.55	16.65
Crude carbohydrate	6.69	6.49	6.65
Aspartic acid	6.09	6.04	6.06
Threonine	2.84	2.83	2.83
Serine	2.22	2.19	2.19
Glutamic acid	7.08	7.00	6.99
Proline	1.88	1.92	1.93
Glycine	3.16	3.13	3.15
Alanine	4.42	4.40	4.42
Valine	3.50	3.49	3.51
Isoleucine	2.71	2.68	2.70
Leucine	4.80	4.75	4.78
Tyrosine	1.90	1.75	1.78
Phenylalanine	2.43	2.41	2.43
Histidine	1.24	1.24	1.25
Lysine	4.33	4.32	4.32
Arginine	3.78	3.71	3.73
Cystein	0.70	0.72	0.71
Methionine	1.64	1.69	1.64
Tryptophan	0.70	0.65	0.67

[a]Compositions in g per 100 g dried biomass.

Supplementary Table 3. Raw data for the *Bacillus subtilis* 168 Δ*sigF-spoVAF* biomass composition presented in Supplementary Table 1[a]

Component (%)	#1	#2	#3
Water	2.07	2.09	2.08
Crude protein	70.13	70.52	70.22
Crude fat	5.06	5.57	5.22
Ash	15.35	15.75	15.55
Crude carbohydrate	7.39	6.07	6.93
Aspartic acid	6.29	6.36	6.40
Threonine	2.54	2.56	2.59
Serine	1.96	1.96	1.99
Glutamic acid	9.81	9.85	10.02
Proline	1.50	1.51	1.51
Glycine	2.42	2.44	2.44
Alanine	4.12	4.11	4.16
Valine	4.20	4.20	4.22
Isoleucine	2.68	2.73	2.71
Leucine	9.55	9.68	9.67
Tyrosine	1.52	1.51	1.44
Phenylalanine	2.05	2.07	2.07
Histidine	1.05	1.07	1.07
Lysine	4.00	4.05	4.05
Arginine	2.64	2.64	2.63
Cystein	0.41	0.42	0.44
Methionine	1.45	1.50	1.54
Tryptophan	0.40	0.37	0.36

[a]Compositions in g per 100 g dried biomass.

Supplementary Table 4. Microbial strains used in this study

Microbial strain	Description	Source[a]
EcN	Probiotic *Escherichia coli* Nissle 1917	Lab stock
168 ΔsigF-spoVAF	Prototrophic and sporulation-defective (Δ*sigF-spoVAF*) mutant of *Bacillus subtilis* 168	Lab stock
KCTC 7913	Crabtree-positive, polyploid, auxotrophic yeast strain *Saccharomyces cerevisiae* KCTC 7913	KCTC
KCCM 50045	Crabree-negative, prototrophic yeast strain *Cyberlindnera jadinii* KCCM 50045	KCCM

[a]KCCM, Korean Culture Center of Microorganisms; KCTC, Korean Collection for Type Cultures

Supplementary Table 5. Composition of MR medium (pH7.0)

Component	Concentration (per L)	
	MR	Modified YM
Citric acid (Sigma-Aldrich, Germany)†	0.8 g	-
Trace metal solution†	5 mL	-
KH_2PO_4 (Junsei, Japan)†	6.67 g	-
$(NH_4)_2HPO_4$ (Junsei, Japan)†	4 g	-
KOH (Junsei, Japan)†	Adjust pH to 7.0	-
Glucose (Samyang, Republic of Korea)‡	20 g	20 g
$MgSO_4 \cdot 7H_2O$ (Junsei, Japan)‡	0.8 g	-
Yeast extract (Gibco, USA)†	-	3 g
Malt extract (BD DIFCO, USA)†	-	3 g
Peptone (Gibco, USA)†	-	5 g

†Autoclaved together as a concentrated solution.

‡Autoclaved or filtered separately as stock solutions and added after cooling.

Supplementary Table 6. Composition of trace metal solution for MR medium

Component	Concentration (per L)
Concentrated HCl (Junsei, Japan)	5.0 mL
$CaCl_2$ (Sigma-Aldrich, Germany)	2.0 g
$FeSO_4 \cdot 7H_2O$ (Junsei, Japan)	10.0 g
$ZnSO_4 \cdot 7H_2O$ (Sigma-Aldrich, Germany)	2.2 g
$MnSO_4 \cdot 5H_2O$ (Junsei, Japan)	0.58 g
$CuSO_4 \cdot 5H_2O$ (Sigma-Aldrich, Germany)	1.0 g
$(NH_4)_6Mo_7O_{24} \cdot 4H_2O$ (Junsei, Japan)	0.10 g
$Na_2B_4O_7 \cdot 10H_2O$ (Sigma-Aldrich, Germany)	0.02 g

Supplementary Notes

Supplementary Note 1. Increasing demands on sustainable non-animal egg substitutes.

In recent years, the demand for egg substitutes derived from non-animal sources has gained momentum. This shift is driven not only by ethical and environmental concerns associated with traditional poultry farming but also by economic factors that make the egg supply vulnerable[2]. Traditional egg production is resource-intensive, requiring significant amounts of water, feed, and land, and contributes to deforestation and biodiversity loss[2]. Additionally, this sector is a notable source of greenhouse gas emissions, including methane and nitrous oxide from manure management, and faces challenges with waste disposal that can lead to water pollution and eutrophication[2]. The routine use of antibiotics to promote growth of chicken and prevent disease exacerbates public health risks by fostering antibiotic-resistant bacteria[3]. Moreover, issues such as avian influenza outbreaks and the fluctuating costs of feed have led to sudden spikes in egg prices, raising food security concerns and highlighting the fragility of reliance on animal-based food systems. These factors underline the pressing need for sustainable egg alternatives that can satisfy consumer demands for nutritional security, culinary diversity, and ethical consumption practices, without contributing to the environmental challenges posed by poultry farming.

Supplementary Note 2. Assessing mechanical properties of the heat-treated microbial lysates and liquid eggs.

Cylindrical specimens with a diameter of 8.5 mm were prepared for compression test by incubating the microbial cell lysates or liquid eggs in 2-mL microcentrifuge tubes at 100 °C for 1 h and cutting into 10-mm long fragments. Heat-treated *S. cerevisiae* lysate was excluded from the compression test since intact specimens could not be prepared due to the heat-induced CO_2 evolution and subsequent cavity formation (Fig. 1a).

The mechanical properties of diluted egg white samples could not be examined since the diluted samples attached to the mould after the heat treatment and hence intact specimens could not be displaced from the mould (Supplementary Fig. 11). In addition, the mechanical properties of diluted egg yolk samples were not evaluated because the undiluted egg yolk showed compression profiles quite distinct from the heat-treated microbial cell lysates as well as egg white and whole egg (Table 1, Supplementary Figs. 1–4 and 9–10).

Supplementary References

1. Ouedraogo, N., Savadogo, A., Somda, M. K., Zongo, C. & Traore, A. S. Effect of mineral salts and nitrogen source on yeast (Candida utilis NOY1) biomass production using tubers wastes. *Afr J Biotechnol* **16**, 359-365 (2017).
2. Guillaume, A., Hubatová-Vacková, A. & Kočí, V. Environmental impacts of egg production from a life cycle perspective. *Agriculture* **12**, 355 (2022).
3. Lima, É., Oliveira, M. B. & Freitas, A. Antibiotics in intensive egg production: Food safety tools to ensure regulatory compliance. *Food Chem Adv* **3**, 100548 (2023). https://doi.org/https://doi.org/10.1016/j.focha.2023.100548
